# Role of lymphangiogenesis in epithelial ovarian cancer

**DOI:** 10.1038/sj.bjc.6603144

**Published:** 2006-05-09

**Authors:** S S Sundar, H Zhang, P Brown, S Manek, C Han, K Kaur, M F L Charnock, D Jackson, T S Ganesan

**Affiliations:** 1 Ovarian Cancer Group, Cancer Research UK Molecular Oncology Laboratories, Weatherall Institute of Molecular Medicine, John Radcliffe Hospital, Oxford OX3 9DS, UK; 2 Department of Cellular Pathology, John Radcliffe Hospital, Oxford OX3 9DS, UK; 3 Cancer Research UK, Churchill Hospital, Headington, Oxford OX3 7LF, UK; 4 Department of Obstetrics and Gynaecology, John Radcliffe Hospital, Headington, Oxford OX3 9DS, UK; 5 MRC Human Immunology Unit, Weatherall Institute of Molecular Medicine, John Radcliffe Hospital, Headington, Oxford OX3 9DS, UK

**Keywords:** lymphatic density, vascular density, ovarian carcinoma, angiogenesis, lymphangiogenesis

## Abstract

We investigated the significance of lymphatic count, vascular count and angiogenic growth factors using immunohistochemistry in 108 tumour specimens of epithelial ovarian cancer with antibodies to lymphatic vessel endothelial hyaluronan receptor (LYVE-1), platelet endothelial cell adhesion molecule CD31, vascular endothelial growth factor (VEGF) and thymidine phosphorylase (TP) in epithelial ovarian cancer to understand the pathogenesis of metastasis in ovarian cancer. The effect of prognostic variables on progression-free and overall survival was assessed. On multivariate analysis, bulky residual disease after surgery was the best prognostic indicator (*P*<0.001) for progression-free and overall survival (*P*<0.001). Lymphatic count was statistically significant as a prognostic factor for progression-free (*P*=0.05) and overall survival (*P*=0.04). However, lymphatic count did not impact on survival curves. No correlation was found between lymphatic count and age, histological subtype, FIGO stage or residual disease. Vascular count, VEGF or TP expressions were not significant in either analysis. Lymphatic spread may be significant in aiding metastases in ovarian cancer but requires other biological factors to act in conjunction, as it does not have clearcut prognostic significance. Dissemination of ovarian cancer does not occur primarily through vascular or lymphatic routes but may occur through direct intraperitoneal spread of disease.

Epithelial ovarian cancer has an annual incidence of 5000 women in the United Kingdom and is the most important cause of gynaecological cancer-related mortality in the Western world. Owing to the paucity of symptoms and their insidious onset, most women present with advanced disease and 5-year survival rates are approximately 30% (Agarwal and Kaye, 2003). Management of ovarian cancer in most patients involves surgery to achieve surgical cytoreduction followed by chemotherapy as surgical cytoreduction highly correlates with better patient survival (Cannistra, 2004). Residual disease of more than 2 cm has traditionally been associated with worse outcome. Sixty to seventy per cent of patients initially respond to platinum-based chemotherapy and approximately 40–50% achieve a complete clinical remission. However, even in this latter group at least half of the patients experience a recurrence within 4 years. Treatment following relapse after initial chemotherapy is palliative in intent.

It has long been known that some cancers metastasise through lymphatics to the regional lymphnodes before widespread dissemination. This progression from locally confined disease to lymph node spread confers a worse prognosis for the patient. Lymphangiogenesis, that is, the formation of new lymphatic vessels may play a role in this (Skobe *et al*, 2001; Stacker *et al*, 2001; Beasley *et al*, 2002). Animal models suggest that lymphangiogenesis occurs in malignancy and that inhibiting this process can halt the spread to lymphatics (Makinen *et al*, 2001; Mandriota *et al*, 2001; Shimizu *et al*, 2004). However, tumour cell invasion of pre-existing lymphatics at the tumour margin can also occur (Jackson *et al*, 2001; Pepper, 2001). Proliferation of lymph vessels is regulated by members of the vascular endothelial growth factor (VEGF) family and their receptors (Oh *et al*, 1997; Shimizu *et al*, 2004). VEGF-C and VEGF-D have been identified as stimulators of lymphatic endothelial proliferation acting on VEGF receptor-3, which functions as a specific receptor in adult tissues (Kaipainen *et al*, 1995). Overexpression of VEGF C and VEGF D in orthotopically transplanted breast carcinoma cells in mice or in VEGF C transgenic mice has been shown to promote tumour lymphangiogenesis and subsequent lymph node metastasis (Pepper, 2001; Skobe *et al*, 2001; Stacker *et al*, 2001).

Understanding the role of lymphatics in human cancers has been improved by the recent identification of a number of novel lymphatic receptors – lymphatic vessel endothelial hyaluronan receptor (LYVE-1), Prox 1, podoplanin, *β*-chemokine receptor D6, macrophage mannose receptor, desmoplakin that can discriminate lymphatic vessels from blood vessels (Knudson and Knudson, 1993; Breiteneder-Geleff *et al*, 1999; Wigle *et al*, 2002; Scavelli *et al*, 2004). LYVE-1, one of the best studied lymphatic markers is a lymph specific receptor for Hyaluronan (HA) (Banerji *et al*, 1999) an abundant extracellular matrix glycosylaminoglycan which facilitates cell migration in wound healing, tumour metastasis and inflammation by forming a pericellular matrix surrounding fibroblasts and epithelial cells, reducing the level of intercellular adhesion (Knudson and Knudson, 1993; Knudson *et al*, 1993). LYVE-1 sequesters HA on lymph vessel endothelium, colocalises with HA on the luminal surface of lymphatic vessels and binds both soluble and immobilised HA exclusively (Prevo *et al*, 2001). LYVE-1 staining of lymphatics is demonstrated in virtually every tissue where these structures could be distinguished and includes vessels draining gastrointestinal tract, skin, lymph nodes, breast and salivary gland and is distinct from blood vessel staining (Banerji *et al*, 1999). However, LYVE-1 is also expressed in normal liver blood sinusoids in mice and humans (Mouta Carreira *et al*, 2001).

There is substantial crosstalk between angiogenesis and lymphangiogenesis in tumour progression as evidenced by the involvement of lymphangiogenic factors VEGF-C, D and their receptor VEGFR3 in angiogenesis and the role played by angiogenic factors VEGF-A and Angiopoetin 2 in lymphangiogenesis respectively (Scavelli *et al*, 2004). Vascular endothelial growth factor is an endothelial cell-specific growth factor and the principal regulator of angiogenesis under normal and pathological conditions in most organs (Ferrara and Alitalo, 1999). Bevacizumab, a VEGF-neutralising antibody has been shown to improve survival in advanced colorectal cancer (Kabbinavar *et al*, 2005). Thymidine phosphorylase (TP) is often induced in the tumour microenvironment by physiological and chemical stress where it protects cells from apoptosis and helps cell survival by stimulating nucleoside metabolism and angiogenesis (Toi *et al*, 2005). The degree of angiogenesis of a tumour as measured by vascular density has emerged as a powerful candidate for prognosis and as a predictive tool in breast cancer and melanomas (Bamberger and Perrett, 2002; Uzzan *et al*, 2004).

This study investigates the significance of tumour lymphatic count and vascular count as well as angiogenic factors VEGF and TP in epithelial ovarian malignancy as prognostic variables to understand the pathogenesis of metastasis in ovarian cancer.

## MATERIALS AND METHODS

### Patient data

The clinical data for this study was collected from unselected patients diagnosed with epithelial ovarian carcinoma between 1990 and 1998 who underwent surgery and treatment at the Oxford Radcliffe Hospitals NHS trust (Oxford, UK). Patients with a histological diagnosis of ovarian carcinoma (borderline tumours were excluded) where follow-up data were known and with sufficient tissue blocks for immunohistochemistry were included. The patients were initially evaluated by clinical, ultrasound examination, computed axial tomography and serum tumour markers. All patients were staged according to FIGO classification (International Federation of Gynaecology and Obstetrics).

Treatment consisted of surgery followed by chemotherapy except in selected patients with Stage 1 disease. Patients who underwent either bilateral salpingoophorectomy with total abdominal hysterectomy and omentectomy or bilateral salpingoophorectomy (where a hysterectomy had been performed previously) were classified as having radical surgery whereas the rest were classified as having debulking surgery to reduce tumour bulk. Residual disease left *in situ* despite surgery was classified as either microscopic, small volume residual disease <2 cm or bulky residual disease >2 cm and information was obtained from the operation summary. The majority of patients who received chemotherapy had platinum-based regimens as first-line treatment. Clinical response data for patients who received chemotherapy was assessed as follows – complete remission was defined as the disappearance of all parameters of the disease; partial remission was defined as a 50% reduction in the size of tumour mass as defined radiologically and progressive disease was defined as a 25% increase of any tumour mass or the development of a new lesion. Overall and progression-free survival were classified as outcome measures. Overall survival was defined as the period from diagnosis until the time of death from any cause or, in patients who were alive, until 1 June 2004. Progression-free survival was calculated as the time period between diagnosis and relapse of disease. Follow-up data were compiled until 1 June 2004.

Blocks from 93 patients were analysed for determination of lymphatic count and 88 patients for vascular count and angiogenic growth factors with some overlap between the two sets, that is, 74 patients had all parameters assessed. Clinicopathological data are presented from a total of 108 patients. Funding organisations approved but did not influence the conduct of the study. Formal consent was obtained to conduct the study from the hospital research and ethics committee.

### Immunohistochemistry

Representative blocks of ovarian cancer with no normal tissue and no large necrotic areas were selected. Three micrometre sections of formalin-fixed, paraffin-embedded tissue were cut onto glass slides and dried. Sections were dewaxed in xylene, rehydrated through alcohol baths and rinsed in water.

### Staining for lymphatic vessel endothelial hyaluronan receptor

For LYVE-1 staining, slides were then pressure cooked for 2 min at full pressure in 0.1 M citrate buffer (pH 6.0) and rinsed in tap water. The primary antibody (rabbit polyclonal anti-human LYVE-1) was incubated for 60 min at a 1/400 dilution, followed by 60 min incubation with the envision polymer (Banerji *et al*, 1999). The slides were then stained on a Dako Techmate500™ using Dako EnVision™ Detection kit Peroxidase/DAB, rabbit/mouse (Dako UK Ltd, Cambridgeshire, UK CB74E1 cat # K5007). Paraffin sections of normal human small intestine were used as a positive control; slides incubated without primary antibody were used as a negative control.

### Immunostaining for platelet endothelial cell adhesion molecule

The JC70 monoclonal antibody, recognizing the pan-endothelial antigen (platelet cell adhesion molecule, PECAM-1) was used as described previously (Horak *et al*, 1992). Epitope retrieval was performed by heat section in microwave for 30 min in tris-ethylenediaminetetraacetate (EDTA) buffer, and blocked in normal human serum for 30 minutes. Slides were incubated with primary antibody JC70 (Dako, mouse monoclonal, Anti-Human CD31, 1 : 50) for 1 h at room temperature. Horse anti-Mouse IgG at 1 : 100 for 30 min at room temperature was used as secondary antibody and the slides were developed using diluted horseradish peroxidase (HRP)–Streptavidin. A tissue section of breast carcinoma previously known to demonstrate strong staining was used as positive control; slides incubated without primary antibody were used as a negative control.

### Immunostaining for vascular endothelial growth factor and thymidine phosphorylase

Antigen retrieval was performed using pressure-cooking for 3 min in Tris-EDTA (pH 9) for VEGF, endogenous peroxidase was then quenched with Dako peroxidase block solution for 5 min. The primary antibody (murine monoclonal antibody VG76e/d9) was applied, rinsed in phosphate-buffered saline and then developed using the HRP envision system (Dako), as described previously (Turley *et al*, 1998). Sections were counterstained with haematoxylin and mounted.

For TP immunostaining, no antigen retrieval was performed and was as described previously (Fox *et al*, 1995). The primary antibody (mouse monoclonal antibody PGF 44-C) was applied as undiluted supernatant for 30 min at room temperature. After washing in tris-buffered saline, sections were treated with rabbit anti-mouse antibody (Dako) diluted 1 in 50 for 30 min and then mouse APAAP complex, 1 in 1, for 30 min. The colour was developed after 15 min incubation with new fuschsin solution. For VEGF, a section of breast carcinoma previously known to demonstrate strong staining was used as a positive control, for TP tumour-associated macrophages were used as an internal positive control, slides incubated without primary antibody were used as a negative control.

### Quantitative analysis of lymphatic vessel, blood vessel density and angiogenic growth factors

Only vessels with typical irregular morphology and a lumen that stained with LYVE-1 antibody were considered lymphatic vessels as some background staining of macrophages and tumour was noted in some sections. The analysis of lymphatic vessel density (LVD) was not restricted to vessels of any specific diameter as per previous papers (Beasley *et al*, 2002; Williams *et al*, 2003). For the microvessel count, small clusters of endothelial cells, with or without a lumen, were considered as individual vessels; single-stained endothelial cells were excluded. Microvessel density (MVD) and LVD were determined in tumour vessel ‘hotspots’. The slides were scanned at low magnification (at × 10 and × 40 for lymphatic vessel and microvessel count respectively) and areas of highest lymphatic vessel or MVD were identified as ‘hotspots’. Each hotspot was then viewed at high magnification using a 0.25 mm^2^ microscope ocular grid (at × 100 and × 400 for lymphatic and microvessel, respectively) and the number of vessels counted in five high-power fields per hotspot. This approach has been validated in published literature (Fox and Harris, 2004). The mean of the vessels in three hotspots per slide was recorded. Highest vessel density was also measured. As advanced ovarian cancer penetrates the ovarian capsule, for the purposes of this study no difference was made between intratumoral and peritumoral lymphatics (Vermeulen *et al*, 2002).

Staining for vascular endothelial growth factor was seen in epithelial, macrophage, stromal and vascular tissue and for TP in epithelial and stromal tissue. Expression was assessed by grading each section on staining intensity with subjective scores ranging from 0 to 3, 0 – negative, 1 – weak, 2– moderate and 3 – strong expression as per previous published studies (Van der Auwera *et al*, 2004). For VEGF, a cumulative score from the four sites of staining was calculated and stratified for analysis (0–3, 4–7, 8–12). The counts were assessed by an operator without prior knowledge of patient data and verified by an independent observer. The two investigators agreed the count on most of the cases. Cases in disagreement were jointly reviewed under a multiheaded microscope and the consensus count was used for analyses.

### Statistics

The Cox proportional hazards model in the Stata statistical program was used to evaluate the putative prognostic indicators considered (age, stage, residual disease, histological type, grade, LVD and vascular density) and to assess any impact on the outcome measures overall and progression-free survival (Cox and Oakes, 1984). Univariate analysis is presented for all these factors. Factors that were statistically significant on univariate analysis were entered into a model to evaluate the prognostic impact on outcome. For multivariate analysis, individual models with either stage or residual disease were computed as both these variables are closely linked, that is, patients with advanced stage disease are more likely to have bulky residual disease after surgery. We also present survival curves using the method of Kaplan and Meier (Kaplan and Meier, 1958).

## RESULTS

### Patients

Clinicopathological data are presented for 108 patients (Table 1). The median overall and progression-free survival was 28.44 and 21 months, respectively. Follow-up information was obtained from 1990 until June 2004 giving a median follow-up of 28.5 months and a range of 0.49–165.29 months. A representative mix of histologies and differentiation was noted in our data set. There was a roughly equal distribution of early (Stage I and II) and advanced stage (Stage III and IV) cancers, which is representative of practice at Oxford. After initial surgery, 49% had microscopic disease, 24% had residual disease ⩽2 cm and 23% had residual disease >2 cm. Fifty-six per cent of patients received platinum-based chemotherapy, 14% received nonplatinum-based chemotherapy and 30% did not receive chemotherapy. Twenty-three of the 108 patients were alive at the time of analysis.

### Evaluation of staining

**Figure 1:**
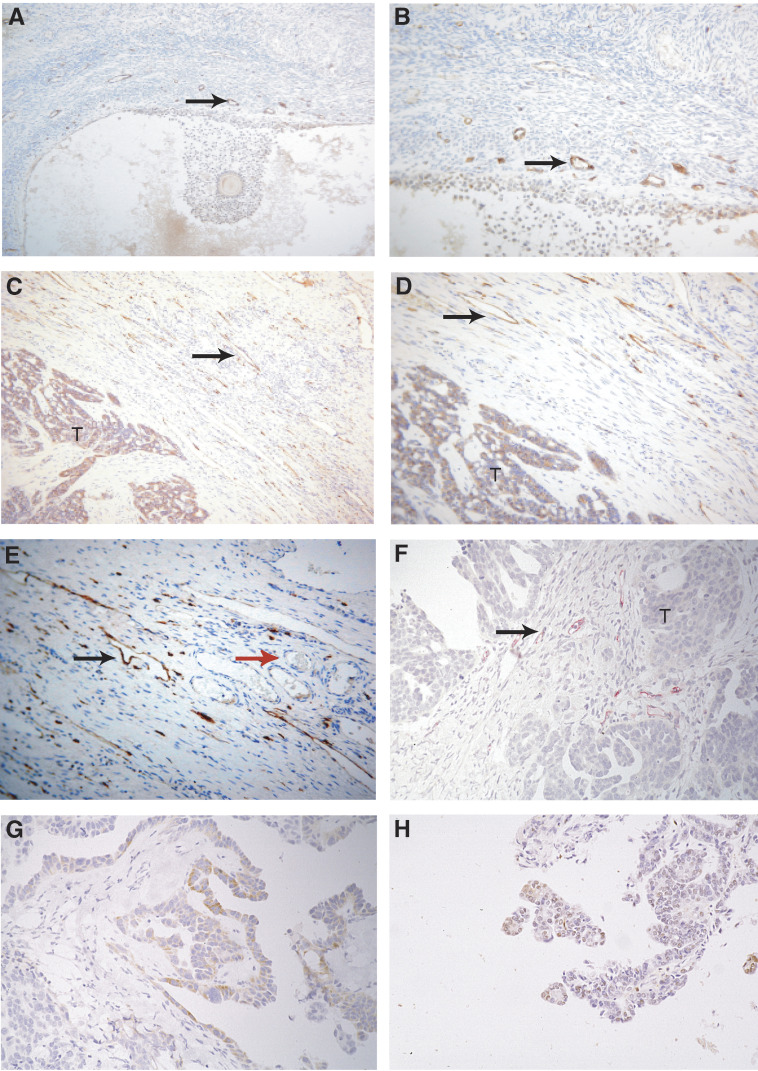
Immunostaining of normal ovary and epithelial ovarian cancer with antibodies. Perifollicular lymphatics in normal ovary at × 10 and × 20 magnification (**A** and **B**). Lymphatic vessels adjacent to tumour at × 10, × 20 and × 40 (**C**–**E**). Blood vessels containing RBC are not stained with LYVE-1 (marked with red arrow). Blood vessels in tumour at × 20 (**F**). Vascular endothelial growth factor staining of tumour epithelium at × 20 (VEGF) (**G**). Epithelial staining of tumour (TP) at × 20 (**H**). Arrowheads identify vessels and T marks tumour.

All but one tumour specimen stained with LYVE-1 antibody. All tumours stained with antibody to CD31 but VEGF and TP staining was more variable (see Supplementary data Table 1 online). Immunostaining with the antibody to the LYVE-1 HA receptor identified irregularly shaped, thin walled lymphatics in the capsular and intratumoural regions of the tumour with capsular lymphatics being more frequent than intratumoural lymphatics. The lymphatics in a section of normal ovary (Figure 1A and B) and tumour stained LYVE-1 antibody (Figure 1C–E). These were distinguished from adjacent blood vessels, which were surrounded by smooth muscle and were regular in shape. Median lymphatic count was 18 (range 0–57). Roughly 52% of tumours had lymphatic counts below the median, high lymphatic counts (>30) were noted in 16% of serous, 21% of endometroid, 7% of clear cell and 20% of mucinous tumours.

Immunostaining with CD31 identified microvessels (Figure 1F). Median vascular was 18.44 (range 7.6–39.75). A total of 61% of tumours had vascular density counts below the median, high vascular counts (>30) were noted in 0% of serous, 7% of endometroid, 7% clear cell and 27% mucinous tumours (Supplementary data Table 1).

Vascular endothelial growth factor staining was noted in various sites (stromal, macrophage, epithelial and vascular) and were quantified (Figure 1G) whereas TP staining (Figure 1H) was noted in the stroma and epithelium of the ovary. Strong staining of VEGF was noted in 29% (epithelial), 17% (stromal), 43% (macrophage) and 19% (vascular) of tumours. Strong staining with TP was noted in 1% (epithelial) and 31% (stromal) of tumours (Supplementary data Table 1).

### Statistical analysis

In univariate analysis (Table 2), age (*P*<0.001), residual disease (*P*<0.001) and FIGO stage (*P*<0.001) were associated with a significantly shorter overall survival and progression-free survival. In this set of patients, histological type or grade of differentiation did not show impact on progression-free or overall survival. Neither lymphatic nor vascular counts were statistically significant on univariate analysis. An analysis of VEGF staining in various sites and TP did not reveal any significance in univariate analysis.

On multivariate analysis (Table 3) two different mathematical models – one with residual disease and the other with stage were used to determine whether lymphatic density was an independent prognostic factor as residual disease and stage are closely related. When all of the significant prognostic factors were taken into account simultaneously in a Cox proportional hazards model, the amount of residual disease was the strongest independent prognostic indicator for overall survival and progression-free survival (*P*<0.001). An analysis of this data set using the Cox proportional hazards model comprising residual disease revealed that lymphatic density reached statistical significance in progression-free survival (*P*=0.05, hazards ratio (HR)1.00–1.05) and overall survival (*P*=0.04, HR 1.00–1.04). When this analysis was repeated with another mathematical model comprising stage and age, LVD was not shown to be of statistical significance. Multivariate analysis using a model with residual disease indicated that the vascular count was not associated with either overall or progression-free survival. Mann–Whitney test did not reveal any statistical significance for either LVD (*P*=0.10) or MVD (*P*=0.87) for response to treatment. Similarly, expression levels of VEGF and TP were not statistically significant in either overall survival or progression-free survival using either of the two models.

**Figure 2:**
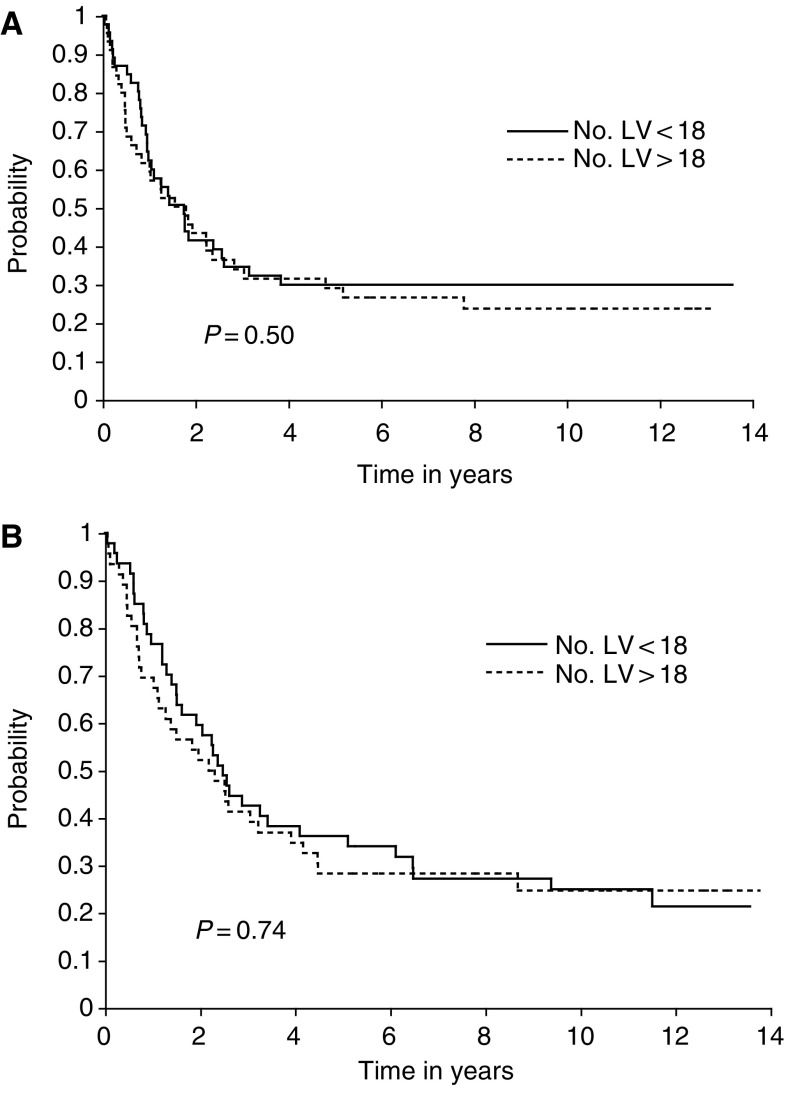
Kaplan–Meier survival plots of lymphatic vessel density in ovarian cancer as an outcome measure for progression-free (**A**) and overall survival (**B**). Lymphatic vessel counts are denoted as < or >18 as indicated. Number of patients=93.

A Kaplan–Meier curve of survival (Figure 2) using a median value of 18 lymphatic vessels as a cutoff did not show any discrimination in survival with high- or low-lymphatic counts. In addition, lymphatic count did not show any correlation with residual disease (Krusker–Wallis *P*=0.53, analysis of variance *P*=0.44) or with histology, differentiation of tumour or vascular count. Lymphatic count did not predict any difference in survival curves for the whole series or in a subset of Stage I patients.

## DISCUSSION

The recent identification of immunohistochemical markers that can reliably distinguish lymphatic vessels from blood vessels in tissue sections has yielded new insight into the mechanisms of metastasis. We investigated LVD, vascular density and angiogenic factors in epithelial ovarian cancer in order to understand the relative contributions of lymphatic and vascular spread in ovarian cancer. This is the first report of LVD in ovarian cancer as measured by LYVE-1, one of the best-characterised markers for lymph vessels.

Our study found that on univariate analysis, both progression-free and overall survival decreased significantly with age, in patients with FIGO stage greater than II and in patients with residual disease after surgery. This is consistent with published literature and clinical practice. In multivariate analysis, using a model adjusting for age and residual disease, LVD was statistically significant in progression-free and overall survival. This was not replicated in another model comprising age and stage of disease. A previous study in epithelial ovarian cancer using a polyclonal antibody to podoplanin (Birner *et al*, 2000) showed that intratumoural LVD was not a statistically significant variable in overall or progression-free survival. This study differed from the previous study in the following ways. We used LYVE-1 as a lymphatic marker. It has been shown previously that these antibodies/markers delineate identical lymphatic vessels on tissue sections (Straume and Akslen, 2004). Secondly, we counted three hotspots as opposed to one hotspot, which may give a more representative view of the number of lymphatic vessels in the tumour. We did not discriminate between intra- and peri-tumoral vessels as ovarian cancer frequently penetrates the ovarian capsule and assessment of LVD as a whole may be relevant in ovarian cancer.

Lymphovascular invasion in tissue sections of tumours has been clearly documented to be a reliable prognostic variable predicting nodal metastasis and survival in breast, cervix cancer and impacts on decision making for therapy in testicular cancer (Warde *et al*, 1997; Morice *et al*, 2003; Schoppmann *et al*, 2004; Dinshaw *et al*, 2005). However, the prognostic significance of LVD in cancer is not clearcut. In head and neck cancer, studies show an association of increased LVD and new vessel formation with lymph node metastasis and worse prognosis (Beasley *et al*, 2002; Maula *et al*, 2003). In melanoma, one large study with 202 samples demonstrated improved overall and recurrence free survival with increased LVD in both peritumoural and intratumoural areas (Straume *et al*, 2003) suggesting that vessel density may be a marker for an improved immune response whereas two smaller studies conclude the opposite with intratumoural lymphatics significantly higher in metastatic melanomas and correlating with poor survival (Dadras *et al*, 2003; Shields *et al*, 2004). Interestingly, in breast cancer intratumoral lymphatic vessels are absent and the significance of peritumoural vessels is unclear (Williams *et al*, 2003; Bono *et al*, 2004; Vleugel *et al*, 2004). Further studies in other tumour types investigating LVD and proliferation markers may clarify the role of new lymph vessel formation and the significance of LVD in cancer progression.

Published studies with blood vessel density in ovarian cancers are equivocal with some studies revealing an association with poor prognosis whereas others dispute this (Hollingsworth *et al*, 1995; Orre *et al*, 1998; Alvarez *et al*, 1999; Abulafia *et al*, 2000; Nakayama *et al*, 2001). We found that MVD in an overlapping set of tumours did not influence prognosis or survival or response to treatment. Both VEGF and TP are important angiogenic factors for many malignancies and have been postulated to work synergistically (Moghaddam *et al*, 1995; Ferrara, 2004). An analysis of the expression of these angiogenic factors in our set of tumours did not reveal any impact on survival. This is similar to some published studies but is in contrast to others (Moghaddam *et al*, 1995; Nishida *et al*, 2004; Raspollini *et al*, 2004; Sonmezer *et al*, 2004).

Lymphatic vessel density in our series was statistically significant in multivariate analysis in overall survival and progression-free survival. Lymphatic counts did not however, discriminate in survival curves. This would suggest that lymphatic spread does not have absolute prognostic significance but may act in conjunction with other biological factors to aid metastasis in ovarian cancer. A larger study of Stage I ovarian cancers will be useful in establishing the prognostic value of LVD and whether this can be used to predict the need for chemotherapy in these patients. Ovarian cancer is primarily an abdominal disease and our study would support the view that intraperitoneal spread of ovarian cancer occurs through direct dissemination of disease rather than through lymphatic or vascular spread.

## Figures and Tables

**Table 1:** Clinicopathological characteristics of the patients

**Characteristic**	**All patients**
Total no.	108
Age	25–88
Median age (years)	63
	
*Histological type*	
Serous	35
Endometrioid	34
Clear cell	16
Mucinous	12
MMMT^a^	4
Mixed epithelial	1
Undifferentiated	1
Primary peritoneal	3
Transitional cell carcinoma	1
Not known	1
	
*Differentiation*	
Poor	29
Moderate–poor	7
Moderate	49
Well	20
Not known	3
	
*FIGO Stage*	
I	32
II	13
III	49
IV	14
	
*Surgery*	
Radical	59
Debulking	46
Not known	3
Residual disease	
Microscopic	53
⩽2 cm	26
>2 cm	25
Not known	4
	
*Chemo-response*	
Complete response	55
Partial response	10
Progressive disease	14
Early death	13
Not evaluable	8
Not known	8
	
*Chemotherapy*	
Platinum	61
Non-platinum	14
No treatment	33

FIGO=International Federation of Gynaecology and Obstetrics.

a Denotes malignant mixed Mullerian tumour.

**Table 2:** Univariate analysis on the association of the variables with the outcome measures

	**Progression-free survival**			**Overall survival**		
**Variable**	**HR**	**95% CI**	***P*-value**	**HR**	**95% CI**	***P*-value**
Age	1.03	1.01–1.05	0.01	1.03	1.01–1.05	0.001
*Residual disease*						
Microscopic	Baseline			Baseline		
⩽2 cm	2.50	1.42–4.40	0.001	2.06	1.20–3.54	0.01
⩾2 cm	7.07	3.94–12.67	<0.001	7.94	4.48–14.08	<0.001
						
*Stage*						
I	Baseline			Baseline		
II	2.14	0.92–4.95	0.08	2.27	1.03–5.04	0.04
III	3.98	2.16–7.34	<0.001	3.78	2.12–6.74	<0.001
IV	6.69	2.82–15.86	<0.001	5.33	2.33–12.15	<0.001
						
Histology						
	Baseline			Baseline		
*Endometrioid*						
Clear cell	0.63	0.31–1.34	0.24	0.65	0.31–1.34	0.24
Mucinous	0.71	0.31–1.65	0.43	0.62	0.28–1.50	0.31
Serous	0.88	0.51–1.53	0.65	1.13	0.68–1.89	0.63
Others	1.25	0.54–2.89	0.60	1.35	0.59–3.11	0.48
						
*Differentiation*						
Well	Baseline			Baseline		
Moderate	1.42	0.73–2.73	0.30	1.32	0.72–2.43	0.37
Moderate–poor	0.77	0.62–5.04	0.29	1.80	0.69–4.69	0.23
Poor	1.92	0.95–3.87	0.07	1.59	0.81–3.12	0.17
						
Lymphatic count	1.01	0.99–1.03	0.30	1.01	0.99–1.03	0.41
Vascular count	0.99	0.95–1.02	0.53	0.98	0.95–1.02	0.34
						
*TP*						
Stroma	1.00	0.81–1.24	0.96	1.11	0.90–1.38	0.33
0/1 *vs* 2 *vs* 3	1.01	0.76–1.35	0.94	1.14	0.85–1.51	0.38
0/1/2 *vs* 3	1.24	0.73–2.08	0.43	1.44	0.86–2.40	0.17
0/1 *vs* 2/3	0.86	0.53–1.41	0.56	1.06	0.65–1.74	0.81
Epithelium	1.21	0.73–2.04	0.46	1.07	0.63–1.80	0.80
						
*VEGF*						
Epithelial	0.97	0.79–1.81	0.74	0.97	0.80–1.19	0.79
Macrophage	1.01	0.84–1.23	0.89	1.02	0.85–1.24	0.81
Stromal	1.01	0.80–1.28	0.94	0.99	0.78–1.26	0.94
Vascular	0.98	0.80–1.20	0.83	0.97	0.79–1.19	0.80
VEGF(0–3,4–7,8–12)	1.05	0.73–1.51	0.79	1.07	0.75–1.54	0.71

CI=confidence intervals; HR=hazards ratios; TP=thymidine phosphorylase; VEGF=vascular endothelial growth factor.

Age was analyzed as a continuous variable. Univariate analysis of TP and VEGF expression was performed by considering the trend in expression.

**Table 3:** Multivariate analysis on the association of the variables with outcome measures. 3A Lymphatic count, 3B Vascular count, VEGF and TP as variables

	**Progression-free survival**			**Overall survival**		
**Variable**	**HR**	**95% CI**	***P*-value**	**HR**	**95% CI**	***P*-value**
(**A**)						
*Model I*						
Age	1.02	1.01–1.05	0.07	1.03	1.01–1.05	0.01
Lymphatic count	1.02	1.00–1.05	0.05	1.02	1.00–1.04	0.04
						
*Residual disease*						
Microscopic	Baseline			Baseline		
⩽2 cm	2.21	1.16–4.20	0.02	1.64	0.088–3.06	0.12
⩾2 cm	7.38	3.77–14.46	<0.001	7.43	3.86–14.31	<0.001
						
*Model II*						
Age	1.02	1.00–1.05	0.05	1.03	1.00–1.05	0.02
Lymphatic count	1.01	0.99–1.03	0.27	1.01	0.99–1.03	0.19
						
*Stage*						
I	Baseline			Baseline		
II	1.57	0.63–3.94	0.34	1.62	0.68–3.84	0.27
III	3.31	1.74–6.31	<0.001	3.05	1.66–5.60	<0.001
IV	6.26	2.63–14.90	<0.001	4.21	1.88–9.68	<0.001
						
(**B**)						
*Model I*						
Age	1.02	0.99–1.04	0.11	1.03	1.01–1.05	0.04
						
*Residual disease*						
Microscopic	Baseline			Baseline		
⩽2 cm	1.93	1.104– 3.58	0.04	1.79	0.97–3.22	0.06
⩾2 cm	6.77	3.58–12.81	<0.001	7.63	4.00–14.58	<0.001
						
Vascular count	0.82	0.48–1.40	0.46	0.77	0.44–1.34	0.35
VEGFe	1.02	0.83–1.25	0.85	1.03	0.84–1.28	0.75
VEGFm	0.99	0.82–1.20	0.93	0.98	0.81–1.19	0.85
VEGFs	1.10	0.86–1.41	0.45	1.07	0.83–1.38	0.59
VEGFv	1.14	0.92–1.42	0.24	1.13	0.91–1.41	0.26
TPs	0.97	0.79–1.20	0.81	1.07	0.86–1.32	0.55
TPe	1.36	0.79–2.33	0.27	1.09	0.63–1.88	0.76
						
*Model II*						
Age	1.01	0.99–1.04	0.34	1.02	0.99–1.04	0.16
						
*Stage*						
I	Baseline			Baseline		
II	2.07	0.85–5.00	0.11	2.25	0.95–5.32	0.07
III	3.41	1.77–6.59	<0.001	3.11	1.61–6.04	0.001
IV	4.44	1.63–12.10	<0.001	4.79	1.74–13.18	0.002
						
Vascular count	0.97	0.57–1.65	0.90	0.95	0.56–1.62	0.85
VEGFe	1.04	0.94–1.29	0.73	1.05	0.84–1.30	0.68
VEGFm	1.01	0.83–1.23	0.90	1.00	0.82–1.22	1.00
VEGFs	1.11	0.87–1.41	0.40	1.06	0.83–1.35	1.00
VEGFv	1.11	0.89–1.39	0.33	1.11	0.89–1.38	0.37
TPs	1.02	0.82–1.26	0.89	1.12	0.90–1.40	0.29
TPe	1.28	0.74–2.19	0.38	0.99	0.57–1.70	0.96

CI=confidence intervals; HR=hazards ratios; TP=thymidine phosphorylase; VEGF=vascular endothelial growth factor.T
