# Supplementary material for: Role of lymphangiogenesis in epithelial ovarian cancer
Source: Br J Cancer. 2006 May 9;94(11):1650–7. doi: 10.1038/sj.bjc.6603144 (PMC2361318; doi:10.1038/sj.bjc.6603144)
Supplement: Supplementary data [file 94-6603144x1.doc]

**Table 2**: Lymphatic vessel density, microvessel density, VEGF and TP expression in tumour according to histological type.

|  | **0 – 9** | **10 – 19** | **20 – 29** | **30+** | **Total** |
| --- | --- | --- | --- | --- | --- |
| LVD n=95 |  |  |  |  |  |
| Serous | 9 | 8 | 8 | 5 | **30** |
| Endometroid | 6 | 9 | 8 | 6 | **29** |
| Clear cell | 1 | 6 | 6 | 1 | **14** |
| Mucinous | 3 | 4 | 1 | 2 | **10** |
| Other |  | 2 | 4 | 4 | **10** |
| **Total** | **19** | **29** | **27** | **18** | **93** |

|  | **0 – 9** | **10 – 19** | **20 – 29** | **30+** | **Total** |
| --- | --- | --- | --- | --- | --- |
| MVD n=88 |  |  |  |  |  |
| Serous | 0 | 17 | 11 | 0 | **28** |
| Endometroid | 1 | 16 | 10 | 2 | **29** |
| Clear cell | 1 | 10 | 1 | 1 | **13** |
| Mucinous | 1 | 4 | 3 | 3 | **11** |
| Other | 0 | 4 | 3 | 0 | **7** |
| **Total** | **3** | **51** | **28** | **6** | **88** |

|  | Epithelial | | | | **Stromal** | | | | **Macrophage** | | | | **Vascular** | | | | **Total** |
| --- | --- | --- | --- | --- | --- | --- | --- | --- | --- | --- | --- | --- | --- | --- | --- | --- | --- |
| VEGF n=88 | **0** | **1** | **2** | **3** | **0** | **1** | **2** | **3** | **0** | **1** | **2** | **3** | **0** | **1** | **2** | **3** |  |
| Serous | 11 | 8 | 0 | 9 | 16 | 5 | 3 | 4 | 6 | 5 | 3 | 14 | 11 | 10 | 2 | 5 | **28** |
| Endometroid | 13 | 4 | 3 | 9 | 20 | 4 | 3 | 2 | 9 | 3 | 4 | 13 | 18 | 4 | 3 | 4 | **29** |
| Clear cell | 6 | 4 | 2 | 1 | 10 | 1 | 0 | 2 | 5 | 2 | 3 | 3 | 8 | 1 | 2 | 2 | **13** |
| Mucinous | 4 | 4 | 0 | 3 | 8 | 2 | 1 | 0 | 3 | 2 | 2 | 4 | 5 | 1 | 2 | 3 | **11** |
| Other | 2 | 1 | 0 | 4 | 3 | 0 | 0 | 4 | 3 | 0 | 0 | 4 | 3 | 0 | 3 | 1 | **7** |
| **Total** | **36** | **21** | **5** | **26** | **57** | **12** | **7** | **12** | **26** | **12** | **12** | **38** | **45** | **16** | **12** | **17** | **88** |

|  | **Epithelial** | | | | **Stromal** | | | | **Total** |
| --- | --- | --- | --- | --- | --- | --- | --- | --- | --- |
| TP n=88 | **0** | **1** | **2** | **3** | **0** | **1** | **2** | **3** |  |
| Serous | 19 | 7 | 2 | 1 | 8 | 5 | 6 | 9 | **28** |
| Endometroid | 22 | 5 | 2 | 0 | 7 | 3 | 9 | 10 | **29** |
| Clear cell | 9 | 2 | 1 | 0 | 3 | 2 | 4 | 4 | **13** |
| Mucinous | 7 | 2 | 2 | 0 | 3 | 4 | 1 | 3 | **11** |
| Other | 5 | 1 | 1 | 0 | 1 | 5 | 0 | 1 | **7** |
| **Total** | **62** | **17** | **8** | **1** | **22** | **19** | **20** | **27** | **88** |

LVD and MVD denote lymphatic vessel and microvessel density respectively.
